# Effect of Nano-SiO_2_ on the Hydration and Microstructure of Portland Cement

**DOI:** 10.3390/nano6120241

**Published:** 2016-12-15

**Authors:** Liguo Wang, Dapeng Zheng, Shupeng Zhang, Hongzhi Cui, Dongxu Li

**Affiliations:** 1 Jiangsu National Synergetic Innovation Center for Advanced Materials (SICAM), Nanjing Tech University, Nanjing 211800, China; wanglg@njtech.edu.cn (L.W.); 815806703@njtech.edu.cn (S.Z.); 2 Guangdong Provincial Key Laboratory of Durability for Marine Civil Engineering, College of Civil Engineering, Shenzhen University, Shenzhen 518060, China; zhengdapeng@student.cumtb.edu.cn

**Keywords:** nano-SiO_2_, cement-based materials, physical and mechanical properties, porosity

## Abstract

This paper systematically studied the modification of cement-based materials by nano-SiO_2_ particles with an average diameter of about 20 nm. In order to obtain the effect of nano-SiO_2_ particles on the mechanical properties, hydration, and pore structure of cement-based materials, adding 1%, 3%, and 5% content of nano-SiO_2_ in cement paste, respectively. The results showed that the reaction of nano-SiO_2_ particles with Ca(OH)_2_ (crystal powder) started within 1 h, and formed C–S–H gel. The reaction speed was faster after aging for three days. The mechanical properties of cement-based materials were improved with the addition of 3% nano-SiO_2_, and the early strength enhancement of test pieces was obvious. Three-day compressive strength increased 33.2%, and 28-day compressive strength increased 18.5%. The exothermic peak of hydration heat of cement increased significantly after the addition of nano-SiO_2_. Appearance time of the exothermic peak was advanced and the total heat release increased. Thermogravimetric-differential scanning calorimetry (TG-DSC) analysis showed that nano-SiO_2_ promoted the formation of C–S–H gel. The results of mercury intrusion porosimetry (MIP) showed that the total porosity of cement paste with 3% nano-SiO_2_ was reduced by 5.51% and 5.4% at three days and 28 days, respectively, compared with the pure cement paste. At the same time, the pore structure of cement paste was optimized, and much-detrimental pores and detrimental pores decreased, while less harmful pores and innocuous pores increased.

## 1. Introduction

Cement-based materials are widely used as building materials around the world. With the development of cement-based materials, its performance is becoming more and more important in modern construction. There are many defects in ordinary cement materials, which will have an adverse impact on the mechanical properties and durability. In recent years, many researchers have conducted many studies on cement modification through adding mineral admixtures in cement, and have achieved significant progress [1,2].

With the development of nanotechnology, its application in cement-based materials has become a research hotspot. Nanomaterials are functional materials with many excellent properties, such as size effects, quantum effects, surface effects, and interfacial effects [3]. These properties can enhance the physical and chemical properties of cement, and open up new areas for cement research. Some researchers used nanomaterial as an additive added into cement-based materials, and have obtained some remarkable results. Recently, many nanomaterials, such as nano-TiO_2_ [4,5,6,7,8], nano-CaCO_3_ [9,10], nano-Al_2_O_3_ [11,12,13], and carbon nano-tubes [14] have been added in cement-based materials to improve various properties of cement. Compared with other nano-materials, nano-SiO_2_ has more advantages, since it has a higher pozzolanic activity. Nano-SiO_2_ has a retardation effect on the cement paste structure, and fills the voids between the cement particles [15]. It was reported that nano-SiO_2_ can promote the hydration of cement and generated more C–S–H gel [16,17]. Researchers have done much work on the influence of nano-SiO_2_ on the hydration and mechanical properties of cement. The Table 1 shows the improvement of properties of cementitious composites with nano-SiO_2_. In addition, many researchers studied the influence of nano-SiO_2_ on the durability of concrete, and the results showed that the suitable content of nano-SiO_2_ can improve the durability of concrete significantly [18,19,20,21].

However, little research has been done on the microstructure and macroscopic properties in the same research. In this paper, the influence of nano-SiO_2_ particles on the hydration, microstructure and mechanical properties of cement was studied systematically by means of hydration heat, X-ray diffraction (XRD), thermogravimetric-differential scanning calorimetry (TG-DSC), scanning electron microscopic (SEM) observation, and mercury intrusion porosimetry (MIP). The impact of nano-SiO_2_ particles on the hydration process and pore structure of cement at early ages was studied emphatically, which will have a certain effect on the influence of nano-SiO_2_ on the hydration mechanism of cement and provide a basis for deep research for future generations.

## 2. Materials and Methods

### 2.1. Materials and Mix Proportions

#### 2.1.1. Materials

The cement used for this experiment was PII 52.5 N ordinary Portland cement supplied by Jiangnan onoda cement Co., Ltd. (Nanjing, China). The physical properties and chemical compositions of Portland cement were showed in Table 2 and Table 3. The standard sand used in this study was obtained from China ISO standard sand Co., Ltd. (Xiamen, China). Nano-SiO_2_ was obtained from Nanjing TANSAIL Advanced Materials Co. Ltd. (Nanjing, China). Characterization of the used nano-SiO_2_ were shown in Table 4. The water used in this study was all local tap water.

**Figure 1:**
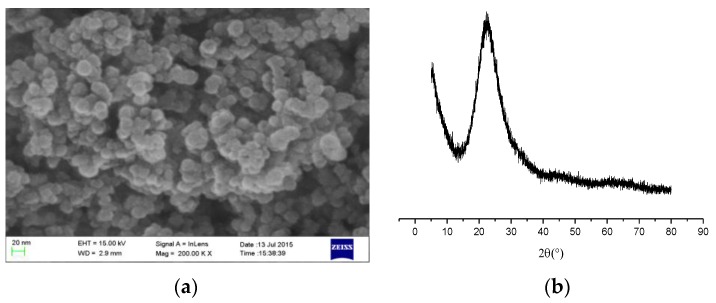
(**a**) Scanning electron microscopic (SEM) patterns of nano-silica; (**b**) X-ray diffraction (XRD) patterns of nano-silica.

The composition and morphology of nano-silica can be analyzed by scanning electron microscopic (SEM) observation and X-ray diffraction (XRD). Nano-silica showed in Figure 1 and the mean particle size of nano-silica was about 20 nm. The samples were scanned from 5° to 85° 2θ at a scanning speed of 10°/min (Figure 1). The crystallization degree of nano-SiO_2_ is very poor which revealed the nano-silica possesses very strong reactive activity.

#### 2.1.2. Sample Preparation for Nano-SiO_2_ and Ca(OH)_2_ Reaction Test

In the system of nano-SiO_2_-Ca(OH)_2_-H_2_O, Ca(OH)_2_, nano-SiO_2_, and water mass ratio were shown in Table 5. Nano-SiO_2_ and Ca(OH)_2_ powder adequately mixed two times in a mechanical agitator (4000 r/min), and 15 min each time. The preparation of nano-SiO_2_ and Ca(OH)_2_ reaction samples followed GB/T 1346-2001.

#### 2.1.3. Preparation of Cement Mortar

The mix proportions were shown in Table 6. In the system 1 wt %, 3 wt %, and 5 wt % nano-SiO_2_ (by cement mass) was added in cement. In order to keep with the same fluidity of cement paste, 0.13 wt %, 0.26 wt % superplasticizer was adding into cement mortar. In order to solve the problem of aggregation of nano-SiO_2_, the pre-process method of ultrasonic dispersion was adopted. Specific mixing process is as follows: all nano-SiO_2_ and water were mixed first, and using an ultrasonic machine with 50 W power for 5 min. Then superplasticizer was mixed in a mortar mixer with sand and cement for 2 min at low speed. After low speed mixing, the sonicated mixture was added and mixed for 1 min at low speed and 2 min at high speed. Cement mortar was cast in the mold with dimensions of 4 cm × 4 cm × 16 cm immediately after mixing. The specimens were striped after 24 h and cured in water at 20 ± 1 °C for specified ages (3 days, 7 days, and 28 days). Three samples of each mortar type were subjected to flexural and compressive strength tests each time. The basic properties of cement samples tested were based on GB/T 17671-1999.

### 2.2. Testing Procedures

In this experiment, different contents of nano-SiO_2_ were added with molding paste. The resulting cement paste was immediately poured into a 10 mm × 10 mm × 10 mm mold. Then the samples were cured at 20 ± 1 °C. After 24 h, the specimens were removed from the mold and cured at the same condition samples are packed in small bottles with anhydrous ethanol for microscopic tests. Microstructural properties of hydrated cubes were evaluated.

XRD was used to analyze the hydration products phase of cement pastes. A few samples dried at 50 °C for 12 h were crushed and grounded to powder. XRD was performed using a D max/RB diffractometer (Rigaku, Tokyo, Japan) with a copper target, 40 kV, 100 mV. The scan range was 5°–80°, 0.02°/step, 0.4 s/step.

The hydration exothermic rate of each paste was measured by a TAM air calorimeter (TA Instruments Co., New Castle, DE, USA) to assess the effect of nano-SiO_2_ on the hydration of cement paste. The w/c is 0.5, and the hydration exothermic rate within 72 h was tested.

A scanning electron microscope (SEM, JMS-5900, JEOL, Tokyo, Japan) was used to analyze the morphology of cement paste. Small fractured samples at every hydration age were soaked in anhydrous ethanol to stop hydration and dried at 50 °C for 12 h. Then the sample was coated with 20 nm of gold to make it conductive.

Differential thermal analysis (TG-DSC, NETZSCH, ATA409, NETZSCH, Selb, Germany) was used to test the absorption capacity of nano-SiO_2_. In this test, the heating rate is 20 °C/min. The pore sizes of cement samples were tested by mercury intrusion porosimetry (MIP, poremaster-60, Quantachrome, Houston, TX, USA). A few samples dried at 50 °C for 12 h were crushed into 2–5 mm small pieces. The pressure of mercury was fixed at 30,000 psi.

## 3. Results and Discussion

### 3.1. Activity of Nano-SiO_2_

**Figure 2:**
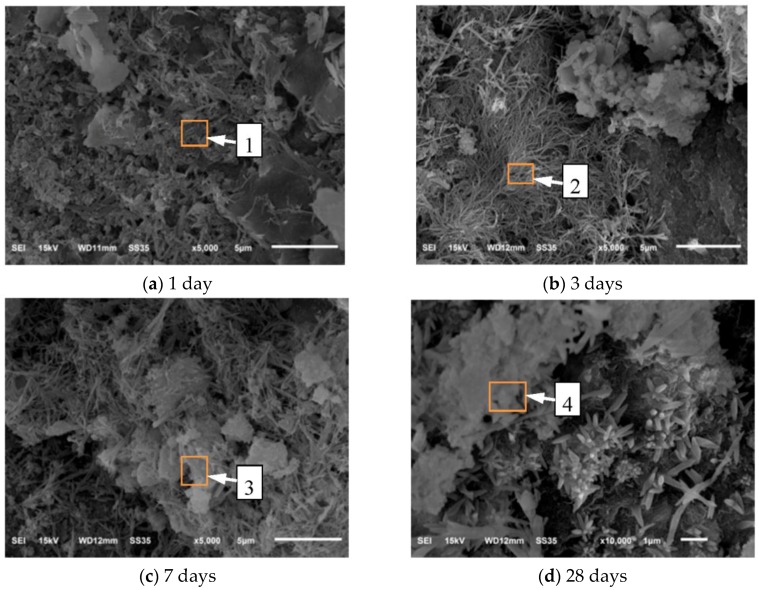
SEM images of microstructure of the products at different ages: (**a**) 1day; (**b**) 3 days; (**c**) 7 days; (**d**) 28 days. 1, 2, 3, 4 are the position numbers in Table 7.

In order to define the microstructure of the reaction product produced by nano-SiO_2_ and Ca(OH)_2_, the corresponding SEM images of the products were also investigated. SEM images of microstructure of the products at different ages were shown in Figure 2. An obvious change can be seen with the increasing ages. Many needle-like and bar-like crystals emerged at the surface of early ages. As the curing age increasing, the resulting flocculated structure tends to become denser. In order to obtain more information of the products, the chemical element of products determined by Energy Dispersive Spectrometer (EDS) and the results were showed in Table 7. It can be seen that the shape and the element percentage of the products was close to C–S–H gel, so we can infer that the production of the reaction was C–S–H gel.

**Figure 3:**
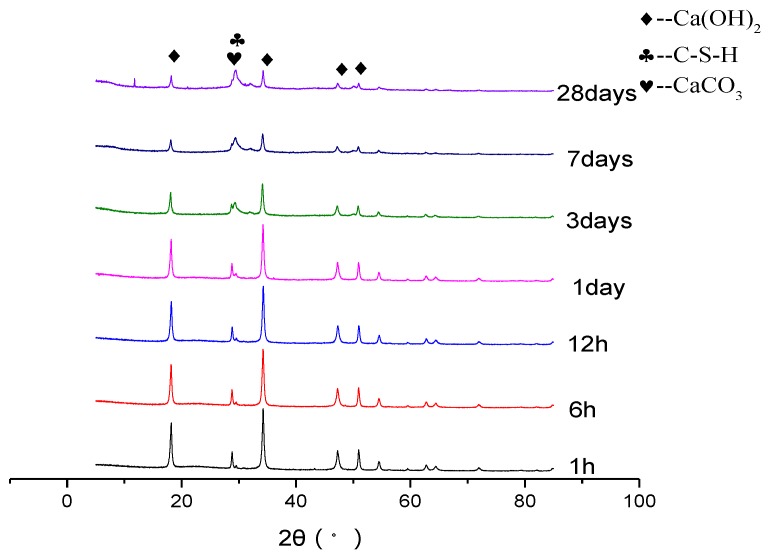
XRD patterns of the reaction of nano-SiO_2_ with Ca(OH)_2_.

XRD analyses were used to investigate the composition of hydration products. Figure 3 shows the XRD analysis of nano-SiO_2_ and Ca(OH)_2_, and we could get the reaction degree of nano-SiO_2_ with Ca(OH)_2_ from it. The nano-SiO_2_ has poor degree of crystallization, so the diffraction peak of Ca(OH)_2_ should be focused and the intensity reflected the contained of Ca(OH)_2_ [4]. It can be seen from Figure 3, the diffraction peak intensity of Ca(OH)_2_ was significantly becoming lower at the ages of 1 h, 6 h, 12 h, one day, three days, seven days, and 28 days, respectively. As it shown in Table 8, the relative diffraction peak intensities at 18° were 100%, 92.12%, 90.47%, 87.73%, 50.84%, 28.69%, and 26.89%, and the relative diffraction peak intensities at 33° were 100%, 92.27%, 91.48%, 89.36%, 50.04%, 29.16%, and 28.78%, respectively. Researchers found diffuse diffraction peaks at 29.1°, 31.8°, 49.8°, and 54.9° in the study of C–S–H synthesis. This was a relatively low Ca/Si C–S–H gel after analysis. We got the main hydration products of nano-SiO_2_ reacted with Ca(OH)_2_, and the characteristics of diffraction peak were similar with the researchers’ results [26]. The C–S–H gel peak existed at 12 h and one day, and became higher at seven days and 28 days. This showed that the reaction of nano-SiO_2_ with Ca(OH)_2_ in cement can occur and form C–S–H gel.

**Figure 4:**
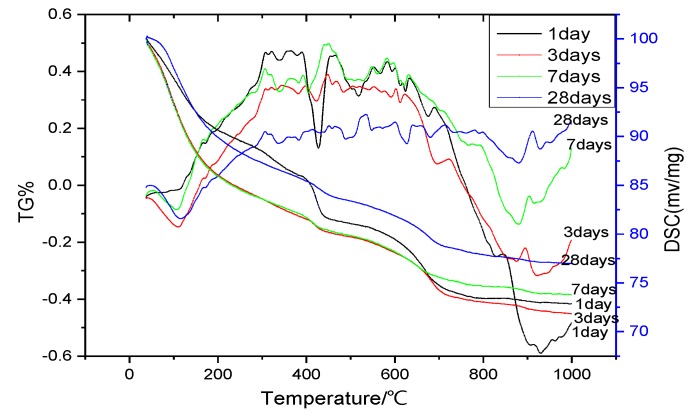
Thermogravimetric-differential scanning calorimetry (TG-DSC) curves of the products of the reaction of nano-SiO_2_ with Ca(OH)_2_.

**Figure 5:**
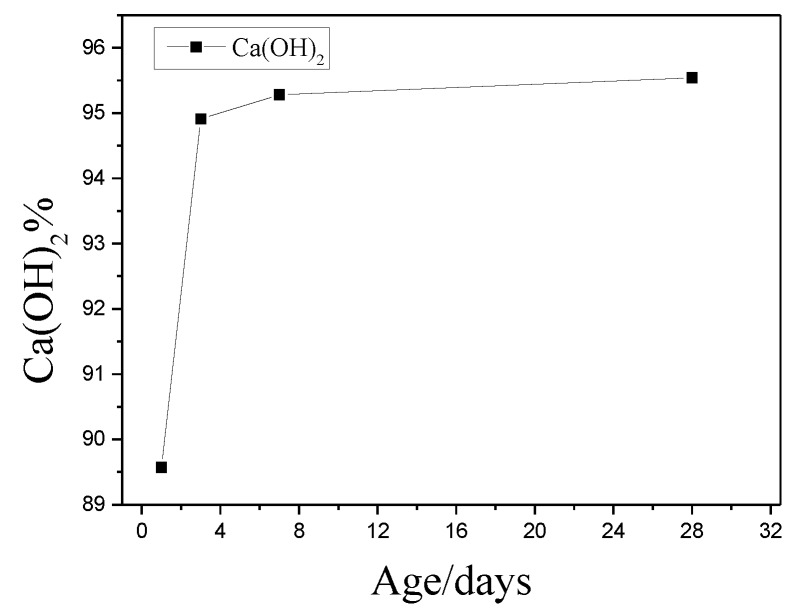
The mass loss of Ca(OH)_2_ at different ages.

The TG-DSC curves for the reaction of nano-SiO_2_ with Ca(OH)_2_ at one day, three days, seven days, and 28 days, respectively, were shown in Figure 4. We could obtain the reaction degree of nano-SiO_2_ with Ca(OH)_2_ by the TG-DSC curves. It can be found that the endothermic peak of Ca(OH)_2_ was most obvious after one day. The endothermic peak of Ca(OH)_2_ was gradually weakened as time went by. Additionally, we can obtain the mass loss of Ca(OH)_2_ based on the TG curves. Ca(OH)_2_ content in the sample by the ratio of the mass loss of sample (Ca(OH)_2_ dehydration peak) to the mass loss Ca(OH)_2_ (analytical reagent) (mass ratio) was estimated. The results are shown in Figure 5. The reaction amounts of Ca(OH)_2_ were 89.57%, 94.91%, 95.28%, and 95.54% at one day, three days, seven days, and 28 days, respectively. The reaction between nano-SiO_2_ and Ca(OH)_2_ was the most intense in one day and the reaction was basically completed in three days.

### 3.2. Compressive and Flexural Strength of Cement Mortar

**Figure 6:**
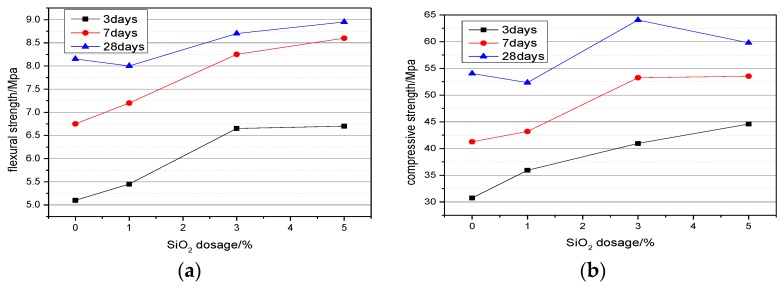
Flexural and compressive strength of cement contained Nano-SiO_2_. (**a**) Flexural strength; and (**b**) compressive strength.

Mechanical properties of all the specimens were measured at different ages. The effect of adding 0%, 1%, 3%, and 5% nano-SiO_2_ on the flexural and compressive strength of cement paste were shown in Figure 6. Compared with the control samples, the flexural strength of cement paste increased 6.9%, 6.7%, and −1.8%, at three days, seven days, and 28 days, respectively, when the content of nano-SiO_2_ was 1%, and compressive strength increased by 16.8%, 4.7%, and −3.2%, respectively. When the content of nano-SiO_2_ was 3%, flexural strength increased by 30.4%, 22.2%, and 6.7% and the compressive strength increased by 33.2%, 29.1%, and 18.5%, respectively. When the dosage of nano-SiO_2_ reaching 5%, flexural strength increased by 31.4%, 27.4%, and 9.8%, and the compressive strength increased by 44.9%, 29.7%, and 10.6% at three days, seven days, and 28 days, respectively. It could be concluded that the higher the content of nano-SiO_2_, the better the early strength of cement. However, it will lead to a slow development of the late strength when the amount of nano-SiO_2_ was too high. Nano-SiO_2_ could provide obvious increases of the strengths both at early and late dates when the dosage of nano-SiO_2_ was 3%. There were reasons for this phenomenon: on the one hand, nano-SiO_2_ had smaller particles, and can fill between the cement particles, so that the density of the paste increased. Nano-SiO_2_ can also be a nucleation point to promote cement hydration and strengthen the connection of cement hydration products [23,27]. On the other hand, nano-SiO_2_ can also react with the hydration product Ca(OH)_2_ to produce more C–S–H gel [15]. We believe that too high or too low content of nano-SiO_2_ are not conducive to the upgrading of cement strength. Zhu [16] also obtained the same results. This may be due to the fact that the quantity of SiO_2_ nano-particles present in the mix is higher than the amount required to combine with the liberated lime during the process of hydration. This leads to excess silica leaching out and causing a deficiency in strength as it replaces part of the cementitious material but does not contribute to its strength [15]. From Figure 6a,b, it can be known that nano-SiO_2_ showed the most obvious enhancement in three-day strength, followed by seven-day strength, and the effect on the strength of 28 day was the weakest.

### 3.3. Effect of Nano-SiO_2_ on the Hydration Heat of Cement

**Figure 7:**
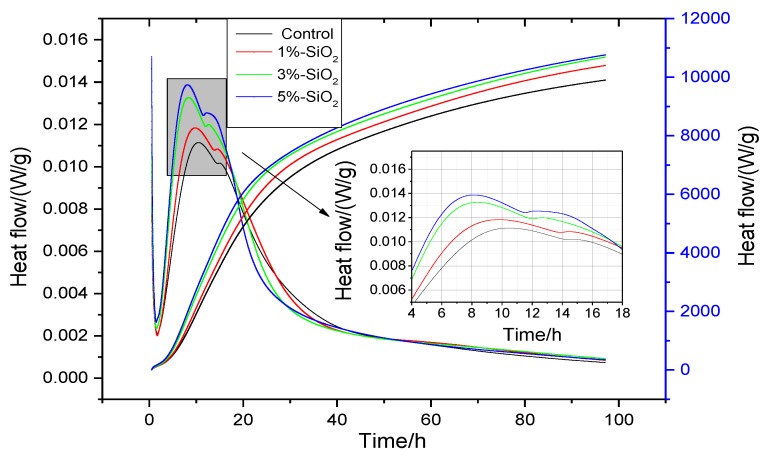
Effect of nano-SiO_2_ on cement hydration exothermic rate and hydration heat.

The hydration exothermic rate of cement paste with 0%, 1%, 3%, and 5% amounts of nano-SiO_2_ within 72 h were shown in Figure 7. There was an obvious difference on heat release compared with pure cement paste.

As we know, the hydration of Portland cement is an exothermic process. It was found that the hydration process of cement with nano-SiO_2_ was similar to Portland cement [17,28]. We found that with the nano-SiO_2_ content increasing, the rate of hydration heat was accelerated. The higher the adding dosage was, the higher hydration rate was, when the ratio was limited in an experiment range, which indicated that nano-SiO_2_ could promote the hydration process of Portland cement.

The second exothermic peak of the sample contained 3% and 5% nano-SiO_2_ appeared at 8 h after water wetting, which were about 0.013 W/g, 0.014 W/g. The second exothermic peak of the standard sample appeared at about 10.5 h after water wetting, and the hydration rate was about 0.011 W/g. Compared with the standard sample, the second exothermic peak appeared about 2.5 h earlier and the heat release rate increased about 0.002 W/g and 0.003 W/g. The heat release rate of sample with 1% Nano-SiO_2_ also increased about 0.001 W/g. This may be related with the high volcanic activity of nano-SiO_2_. Nano-SiO_2_ has a smaller particle size, a greater quantity of atoms distributing on the surface, which resulted in higher chemical activity. Nano-SiO_2_ reacted with Ca(OH)_2_ generated by the hydration of cement, so that Ca(OH)_2_ was consumed and the chemical equilibrium was broken, which promoted the Ca^2+^ arrive supersaturated in advance. Thus, nano-SiO_2_ shorted the induction period and the hydration process was accelerated and the heat release rate advanced. Thus, the induction period, acceleration period, and deceleration phase appeared to advance.

It can be seen from Figure 7, the heat release of cement with nano-SiO_2_ was higher than the pure cement sample. With the increasing of nano-SiO_2_ content, the hydration process was accelerated and the heat release increased significantly. This was consistent with the previous strength trend. The reason may be that the large amounts of nano-SiO_2_ particles depleted Ca(OH)_2_, leading to more crystallized Ca(OH)_2_. The non-hydrated mineral hydration process was accelerated and the accumulation hydration heat of the cement increased.

### 3.4. Effect of Nano-SiO_2_ on Products of Cement Hydration

**Figure 8:**
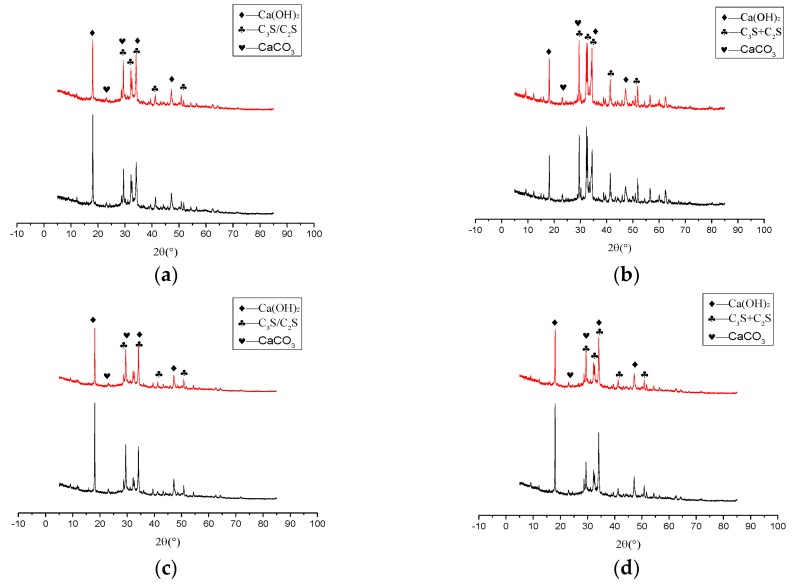
XRD patterns of cement pastes with 3% or without of nano-SiO_2_ at different ages: (**a**) one day; (**b**) three days; (**c**) seven days; and (**d**) 28 days.

To qualitatively evaluate the effect of nano-SiO_2_ on the mineralogy of cement paste, XRD tests were performed and the results were shown in Figure 8. As shown in Figure 6, mixing 3% nano-SiO_2_ in cement made the greatest improvement in mechanical properties of the cement paste, thus, only the sample with 3% nano-SiO_2_ was tested in this part.

It was shown in Figure 8 that the hydration products have not changed after adding 3% nano-SiO_2_ in cement, while the magnitude of the peaks of hydration products changed a lot. The peak intensity of main hydrates variations were shown in Table 9. The magnitude of the intensity of Ca(OH)_2_ at 2θ angle of 18° was tested, showing an increase of 1.42% after one day, but the magnitude of the intensity of Ca(OH)_2_ decreased by 32.24%, 33.31%, and 13.07% at three days, seven days, and 28 days, respectively. This illustrates that more Ca(OH)_2_ was consumed by nano-SiO_2_.

The magnitude of the intensity of the not hydrates such as tricalcium silicate (C_3_S) and dicalcium silicate (C_2_S) was obviously changed. The main peak intensity of C_3_S and C_2_S at a 2θ angle of 33° was also tested. It showed that the peak intensity of C_3_S and C_2_S decreased by 13.08%, 31.40%, 9.08%, and 3.24% at one day, three days, seven days, and 28 days when nano-SiO_2_ was added. It was found that the Ca(OH)_2_ diffraction peak was decreased at the same time, and the C_3_S and C_2_S diffraction peaks also decreased significantly, especially at three days. The results showed that nano-SiO_2_ was reacted with Ca(OH)_2_ and formed C–S–H gel, prompting the hydration reaction to move forward. All of the above results illustrated that nano-SiO_2_ will react with Ca(OH)_2_ and produce the solid C–S–H gel to promote the hydration of C_3_S and accelerated the pace of cement hydration. This conclusion is consistent with the study of Ye [29].

**Figure 9:**
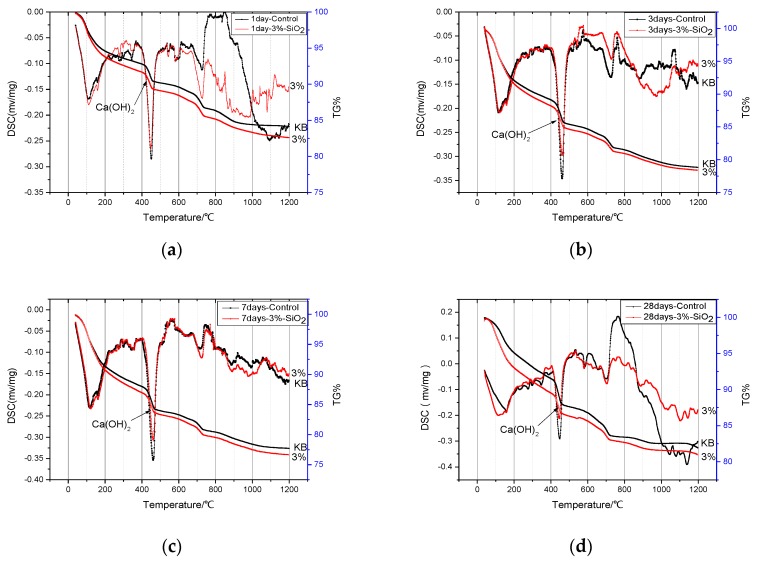
TG-DSC curves of cement pastes with 3% or without of nano-SiO_2_ at different ages: (**a**) 1 day; (**b**) 3 days; (**c**) 7 days; and (**d**) 28 days.

The TG-DSC curves for the cement contained 3% nano-SiO_2_ were shown in Figure 9, which had an obvious difference with the control sample. It can be found that the second endothermic peak of the cement contained 3% nano-SiO_2_ was obviously stronger than the control sample at different ages.

**Figure 10:**
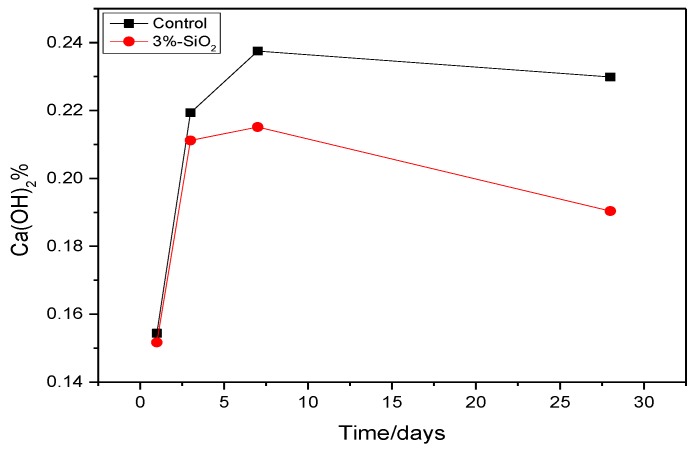
The mass loss of Ca(OH)_2_ at difference ages.

According to the TG curve of cement paste, we can obtain the mass loss of Ca(OH)_2_ at different ages. It can be seen from Figure 10, the content of Ca(OH)_2_ was always lower than that of the control sample with the hydration time increased. The contents of Ca(OH)_2_ in cement paste with 3% nano-SiO_2_ were 0.27%, 0.82%, 2.24%, and 3.95% lower than those in control sample at one day, three days, seven days, and 28 days, respectively. By hydration heat analysis (Figure 7) and XRD analysis (Figure 8), it can be seen that the cement hydration rate was faster at the early age, especially when the nano-SiO_2_ was added to the cement. The hydration promotion of C–S–H by nano-silica mainly occurred at three days of age. However, the Ca(OH)_2_ content in the cement paste did not show any significant decrease after adding nano-SiO_2_ by the TG analysis. This phenomenon meanly because of that the hydration reaction just began at that time and there were much C_3_S and C_2_S which were not hydrated. The hydration reaction of C_3_S was quick and at the same time the pozzolanic activity of nano-SiO_2_ was stronger, and the of Ca(OH)_2_ formed by the hydration reaction of C_3_S and C_2_S reacts with nano-SiO_2_ suddenly. Thus, the production and consumption of Ca(OH)_2_ achieved a dynamic equilibrium, resulting in the Ca(OH)_2_ content tested by TG not decreasing significantly at the three-day age. With the increase of the hydration age, the cement slurry was further hydrated, and the formation of a large number of hydration products, the reaction of nano-SiO_2_ and Ca(OH)_2_ is still continuing, so the content of Ca(OH)_2_ was significantly lower than the control sample.

### 3.5. Effect of Nano-SiO_2_ on Microstructure of the Cement Paste

**Figure 11:**
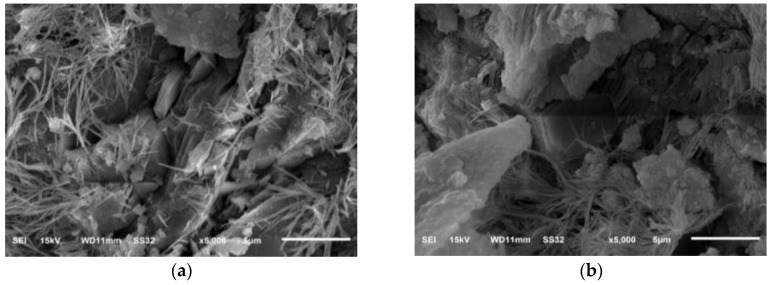

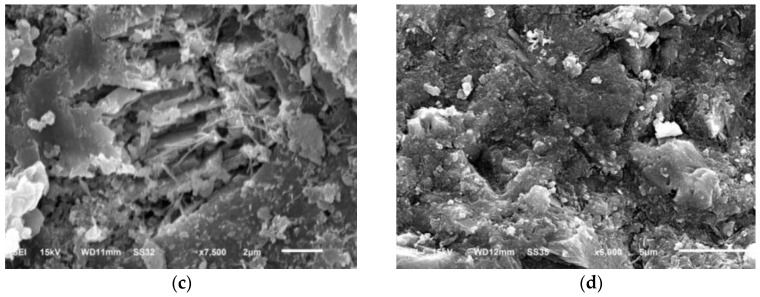
SEM images: (**a**) control cement at three days (**b**) cement with nano-SiO_2_ 3% at three days (**c**) control cement at 28 days; and (**d**) cement with nano-SiO_2_ 3% at 28 days.

Figure 11 showed SEM micrographs of pure cement paste and cement with 3% nano-SiO_2_ at three days and 28 days to directly explore the role of nano-SiO_2_ modifying the properties of cement paste. Figure 11 showed that the sample had a certain development in three days. Figure 11a showed the microstructures existence of needle-hydrates and hexagonal flake of Ca(OH)_2_, but the structure of the cement pastes was very loose with a large number of micron pores. The morphology of C–S–H was non-compact and fibrous. The sample with 3% nano-SiO_2_ was shown in the Figure 11b. As we know, the structural defects can affect the mechanical properties, and comparing with the control sample, the hydration products of the sample with 3% nano-SiO_2_ were different from the control samples. The hydration products became more compact after the addition of nano-SiO_2_, most of the barite ettringite crystals and hexagonal flake of Ca(OH)_2_ had been covered by C–S–H. At 28 days, in Figure 11c, the C–S–H gel with a denser and finer structure of was observed compared with Figure 11a, but the micron pores of the cement paste remained and it had a lot of built-in directional Ca(OH)_2_ crystals embedded in the pores, and each area had relatively independent system. As shown in Figure 11d, the structure of cement paste with 3% nano-SiO_2_ at 28 days was more compact and the Ca(OH)_2_ crystal cannot be found, and the hydration products was much more which have become a whole. Thus, a finer structure formed in the paste, which results in higher strength.

### 3.6. Effect of Nano-SiO_2_ on Pore Structure of Cement Paste

The pore structure of cement paste reflects its compaction rate, which had significant impact on the mechanical property. The more dense the cement paste, the stronger the anti-penetration ability, the stronger the outside corrosion resistance. The pore structure of cement paste can be characterized by the porosity and pore size distribution. According to study of Renhe, et al. [30], the pores in the cement paste can be divided into innocuous pores (the diameter < 20 nm), less harmful pore (20–50 nm), detrimental pores (50–200 nm), and much-detrimental pores (>200 nm). Here, the harmful pores are mainly is respect of the cement-based materials’ mechanical properties and volume stability.

**Figure 12:**
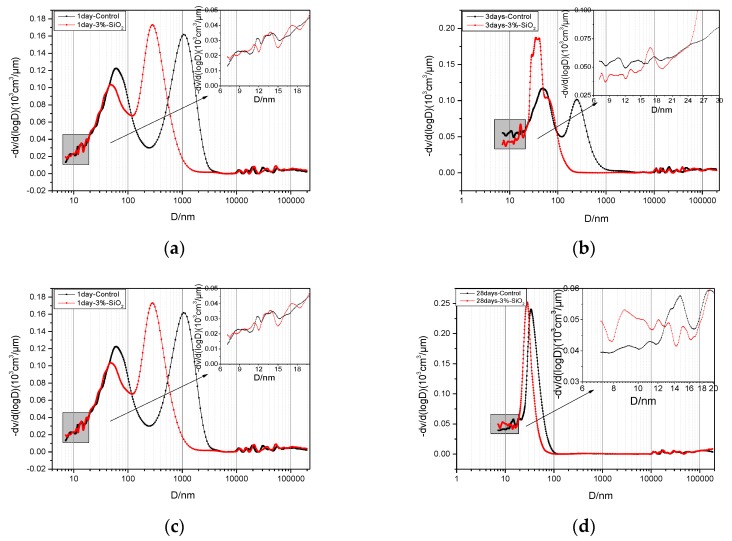
Pore size distribution of cement pastes with 3% or without nano-SiO_2_ at different ages: (**a**) 1 day; (**b**) 3 days; (**c**) 7 days; and (**d**) 28 days.

Figure 12 showed MIP of cement pastes without nano-SiO_2_ and cement paste with 3% nano-SiO_2_ at one day, three days, seven days, and 28 days. As it can be seen from Figure 10, the curves of the entire sample with 3% nano-SiO_2_ had a left shift at different hydration time. This means that the pores size refined and pore structure improved with the addition of nano-SiO_2_.

**Figure 13:**
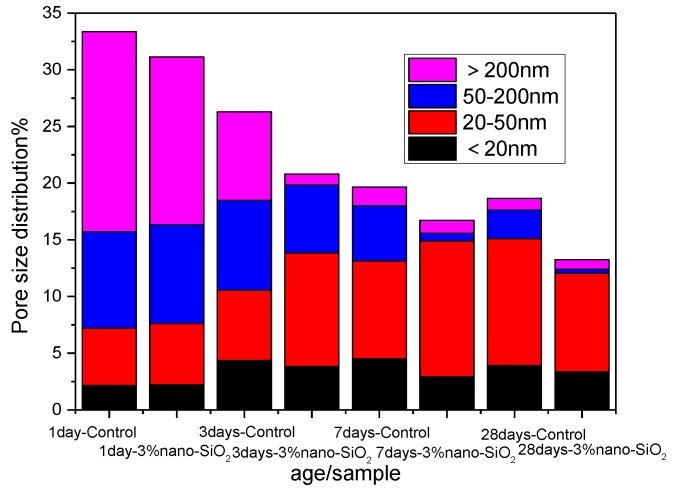
The pore size distribution and porosity of set cement paste with different days.

In order to describe the development of the pore structure of cement paste more accurately, concrete numerical value of porosity of cement pastes at various levels were shown in Figure 13. The total porosity of control sample were 33.35%, 26.30%, 19.67%, and 18.66% at one day, three days, seven days, and 28 days, respectively, while the total porosity of sample with 3% nano-SiO_2_ were 31.14%, 20.79%, 16.72%, and 13.26% respectively. The total porosity of cement paste decreased by 2.21%, 5.51%, 2.95%, and 5.4% at one day, three days, seven days, and 28 days, respectively, after adding 3% nano-SiO_2_. The improvement effect of total porosity is obvious at three days and 28 days. The XRD results showed that the secondary hydration of nano-SiO_2_ and Ca(OH)_2_ accelerates the rate of hydration of C_3_S, resulting in more compact C–S–H gels, increasing the density of the cement paste degree. Porosity improvement is obvious at 28 days; it is mainly due to the hydration of the cement paste, the hydration products become more compact and connected as a whole. The total porosity decreased significantly compared to the early age, and the small particle size of nano-SiO_2_ plays its filling effect, filling between unhydrated particles and voids from further dense cement paste, reducing the total the porosity of cement paste.

The addition of nano-SiO_2_ not only affected the porosity of cement paste, but also affected the pore size distribution at different ages. By analyzing the pore size of different ages in Figure 13, after adding nano-SiO_2_, the porosity of 20–50 nm increased from 15.2% to 17.39%, the percentage of detrimental pores decreased from 52.89% to 47.53% after one day. This was the filling effect of nano-SiO_2_ promoting detrimental pores to transform to less harmful pores. The less harmful pores were increased from 23.59% to 48.23%, and the detrimental pores decreased from 29.78% to 4.65% after three days. The less harmful pores increased from 43.79% to 71.68%, and the detrimental pores decreased from 24.82% to 4.12% after seven days. This was due to the accelerated rate of hydration, and the hydration products filling the pores, making detrimental pores transform to less harmful pores. The detrimental pores decreased from 13.45% to 2.78% and the less harmful pores increased from 20.91% to 25.39% with the continuation of hydration after 28 days.

From the above analysis, it can be concluded that the total porosity of cement paste was effectively reduced and the pore size distribution of cement paste has been effectively improved after adding 3% nano-SiO_2_. The pore structure was optimized by adding nano-SiO_2_, which was not only consistent with the SEM analysis, but also a key factor to improve the mechanical properties of cement mortar at different ages after adding nano-SiO_2_.

## 4. Conclusions

(1) The reaction between nano-SiO_2_ and Ca(OH)_2_ started within 1 h, and the reaction rate was faster in the three day period, and the C–S–H gel was formed.
(2) When the content of nano-SiO_2_ was 3%, the compressive strength increased by 33.2%, 29.1%, and 18.5% at three days, seven days, and 28 days, respectively. The compressive strength increased by 44.9%, 29.7%, and 10.6% at 3 days, 7 days, and 28 days, respectively, when the dosage of nano-SiO_2_ was 5%. Nano-SiO_2_ had the most obvious effect on compressive strength at 3 days, followed by 7 days and 28 days. Taking this into account, 3% was the best dosage of nano-SiO_2_.
(3) Nano-SiO_2_ promoted the hydration heat of cement paste. The effect was obvious when the dosages of nano-SiO_2_ were 3% and 5%, and the heat release rate of hydration heat of the second exothermic peak was increased 0.002 W/g, 0.003 W/g respectively. The second exothermic peak appeared approximately 2.5 h earlier. The cumulative heat release of the paste increased with the adding of nano-SiO_2_.
(4) The content of Ca(OH)_2_ of cement paste with 3% nano-SiO_2_ was decreased by 0.27%, 0.82%, 2.24%, and 3.95% at one day, three days, seven days, and 28 days, respectively. The Ca(OH)_2_ diffraction peak intensity increased by −32.24% and −13.07%, but the tricalcium silicate (C_3_S) and dicalcium silicate (C_2_S) diffraction peak intensity increased by −31.40% and −3.24% at three days and 28 days, respectively. The addition of nano-SiO_2_ promoted the formation of C–S–H gel, and the promotion effect mainly occurred in three days.
(5) The total porosity of cement paste decreased 2.21%, 5.51%, 2.95%, and 5.4% at one day, three days, seven days, and 28 day, respectively, when the dosage of nano-SiO_2_ was 3%. Nano-SiO_2_ optimized the pore structure of cement paste, and much-detrimental pores and detrimental pores decreased while less harmful pores and innocuous pores increased.

## Figures and Tables

**Table 1:** Improvement of properties of cementitious composites with Nano-SiO_2_.

Nano-SiO_2_		Properties (Improvement)		Ages	References
Particle Size	Concentration	Compressive Strength	Porosity		
14 nm	0.5%	25%	-	28 days	Stefanidou [22]
15 nm	1.5%	23.88%	-	90 days	Naji [15]
15 nm	7.5%	12.96%	-	90 days	Hesam [19]
15 + 5 nm	10%	26%	-	28 days	Li [23]
30 nm	2.5%	16%	-	28 days	Zhu [16]
50 nm	6%	29.88%	-	28 days	Najat [24]
80 nm	1.0%	13.71%	-	90 days	Naji [15]
30–100 nm	5%	22.85%	−10.2%	28 days	Kim [25]
120 nm	4%	35.86%	−1.0%	28 days	Yu [17]

**Table 2:** Physical properties of Portland cement.

Type	Density (g/cm^3^)	Surface Area (m^2^/kg)	Normal Consistency (%)	Setting Time/Min	Flexural Strength/MPa			Compressive Strength/MPa	
				Initial	Final	3 days	28 days	3 days	28 days
PII 52.5	3.12	372	0.30	180	260	5.10	8.15	30.75	54.04

**Table 3:** Chemical compositions of Portland cement/wt %.

Type	CaO	SiO_2_	Al_2_O_3_	Fe_2_O_3_	SO_3_	MgO	K_2_O	Ignition Loss
PII 52.5	64.95	18.31	4.21	2.95	4.22	0.64	0.788	3.21

**Table 4:** Characterization of the used nano-silica ^a^.

Type	Appearance	Mean Particle Size (nm)	Purity %	pH	Surface Area (m^2^/g)	Density (g/cm^3^)	Surface Property
TSP-H10	White powder	20	>99.5	4–7	300	0.10	hydrophilic

a Data obtained from the supplier.

**Table 5:** Mix proportions of paste made from Ca(OH)_2_, nano-SiO_2_, and water.

Number	Nano-SiO_2_ (g)	Ca(OH)_2_(g)	Water (g)
A	100	54	230

**Table 6:** Mix proportions of the samples.

Number	Cement (g)	SiO_2_ (%) (by Cement Mass)	Sand (g)	Water (g)	Superplasticizer (%)
1	450	0	1350	225	0
2	450	1	1350	225	0
3	450	3	1350	225	0.13%
4	450	5	1350	225	0.26%

**Table 7:** Chemical element compositions of the products tested by energy dispersive spectrometer (EDS).

Position Number	Atom/%				
	C	O	Si	Ca	Ca/Si
1	30.26	49.49	5.63	14.62	2.60
2	26.09	50.87	8.62	13.64	1.55
3	26.54	52.67	7.41	13.38	1.81
4	19.03	56.26	9.43	15.28	1.62

**Table 8:** Peak intensity of Ca(OH)_2_.

Age	CH (Peak Height) 18	Increment (%)	Relative Diffraction Peak Intensities	CH (Peak Height) 33	Increment (%)	Relative Diffraction Peak Intensities
1 h	2551	0%	100%	3711	0%	100%
6 h	2350	−7.88%	92.12%	3424	−7.73%	92.27%
12 h	2308	−9.53%	90.47%	3395	−8.52%	91.48%
24 h	2238	−12.27%	87.73%	3316	−10.64%	89.36%
3 days	1297	−49.16%	50.84%	1887	−49.96%	50.04%
7 days	732	−71.31%	28.69%	1082	−70.84%	29.16%
28 days	686	−73.11%	26.89%	1068	−71.22%	28.78%

**Table 9:** Peak intensity of Ca(OH)_2_ and C_3_S of samples with or without nano-SiO_2_.

Age	Samples	Intensity (Counts)		Increment (%)	
		CH	C_3_S + C_2_S	CH	C_3_S + C_2_S
1 Day	Control	983	1598	1.42	−13.08
	3% nano-SiO_2_	997	1389		
3 Days	Control	2410	679	−32.24	−31.40
	3% nano-SiO_2_	1633	466		
7 Days	Control	2597	595	−33.31	−9.08
	3% nano-SiO_2_	1732	541		
28 Days	Control	2410	446	−13.07	−3.24
	3% nano-SiO_2_	2095	432		
